# Supplementary figures and images for: Adding MASP1 to the lectin pathway—Leprosy association puzzle: Hints from gene polymorphisms and protein levels
Source: PLoS Negl Trop Dis. 2020 Apr 2;14(4):e0007534. doi: 10.1371/journal.pntd.0007534 (PMC7162614; doi:10.1371/journal.pntd.0007534)

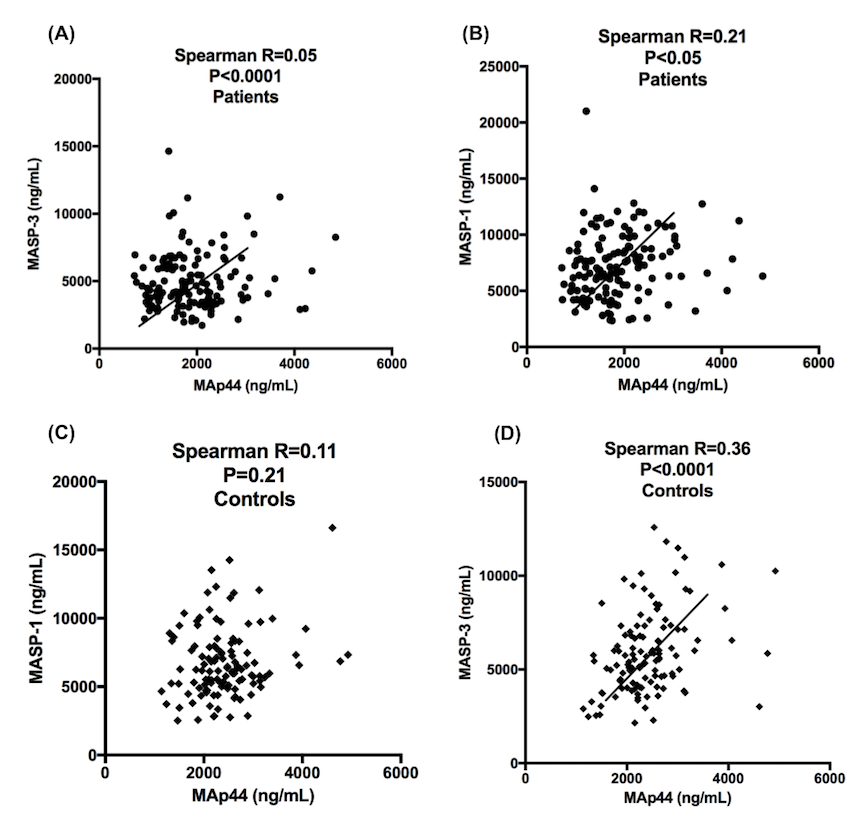

Supplement: S1 Fig — Correlations between MASP-1, MASP-3 and MAp44 serum levels in leprosy patients (A-B) and healthy controls (C-D). Linear regression fit, P and R values are shown. (TIF) [file pntd.0007534.s001.tif]

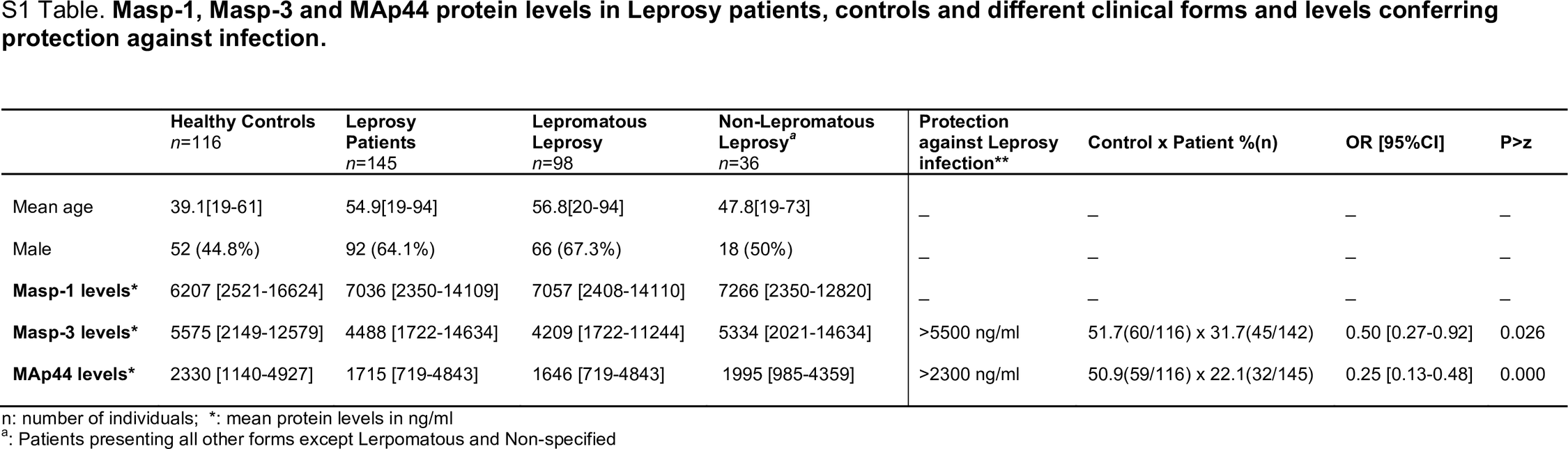

Supplement: S1 Table — n: number of individuals; *: mean protein levels in ug/mL showing: median[IQR] **Levels confering protection against Leprosy infection. Within brackets: minimum and maximal values. a: Patients presenting all other forms except Lepromatous and Non-specified (TIF) [file pntd.0007534.s002.tif]
