# Supplementary material for: Adding MASP1 to the lectin pathway—Leprosy association puzzle: Hints from gene polymorphisms and protein levels
Source: PLoS Negl Trop Dis. 2020 Apr 2;14(4):e0007534. doi: 10.1371/journal.pntd.0007534 (PMC7162614; doi:10.1371/journal.pntd.0007534)
Supplement: Attachment — Submitted filename: Response to reviewers PNTD Weinschutz Mendes 2019.pdf [file pntd.0007534.s004.pdf]

UFPR

To the Editor

Prof. Krithivasan Sankaranarayanan
PLOS Neglected Tropical Diseases

November 8th 2019.

Dear Editor,

Thank you so much for your reply regarding our manuscript entitled Adding MASP1 to the lectin pathway - leprosy association puzzle: hints from gene polymorphisms and protein levels." (#PNTD-D-19-00912). We followed your advices regarding:

1. a list of accession numbers/ID numbers for genes and proteins mentioned added as a paragraph at the end of the manuscript
2. deposition of our PCR-SSP amplification methods in protocols.io - dx.doi.org/10.17504/protocols.io.27kghkw

We also very much appreciated the careful correction and suggestions given by the reviewers. Please find our point-to-point answers below:

Reviewer #2: Overall, the analyses presented match the analysis plan and the results are presented in a clear manner. The figures and tables are okay. However, a couple of clarifications from the authors are necessary:
How do the authors define non-lepromatous, especially w.r.t the numbers given Table 1?
Does non-lepromatous include all the patients who were borderline, indeterminate, and tuberculoid?
Answer: Yes, non-lepromatous includes all patients who presented one of the three forms: borderline, intermediate and tuberculoid. Patients were defined as non-lepromatous if not presenting the lepromatous (multibacillary extreme pole of the spectrum) features. Brazilian health workers are required to enter the clinical characteristics of each leprosy patient into the "SINAN" database (Sistema de Informação de Agravos de Notificação). Each patient file contains information regarding: number and localization of skin patches, neural lesions, eyes, hands and feet disabling degrees, bacilloscopy and histopathology results. Based on these information, patients are diagnosed into one of the clinical forms. As the lepromatous form of leprosy is not only the most disabling, but also the most prevalent in our cohort, we sought to carry out comparisons between this patient group and the less-affected, non-lepromatous individuals. This information was added to the "Subjects and Samples" topic (line 197), thank you for your inquiry.

Can the authors show that the sample sizes within the leprosy cases (for example, 36 non-lepromatous and 97 lepromatous) are sufficient to detect associations with type of leprosy and MASP1 haplotypes/protein levels?
Answer: The power of the allele and haplotype analysis within the patient group was actually too low for detecting small effects. Thus, the absence of genetic association in our study cannot be regarded as definitive. On the other hand, within this same group, the power of the comparison of MASP-3 and Map44 levels reached 70%, increasing our confidence in the results. Even so, the associations detected comparing non-lepromatous and lepromatous patients signalize the importance of further analyses for these clinical forms and its different aspects, they do not draw ultimate conclusions. Other association studies have presented analyses with similar sample sizes (Bene L, et al 2003; Ameye L, et al 2012; Frauenknecht V, et al 2013). Additionally, the sample size for protein quantification analyses represent the percentage of different clinical forms of leprosy usually found in the Brazilian population and due to limitation for protein quantifications (shipping and analyses abroad), the sample size had to be reduced from the total. Part of this discussion was added to the Material and Methods section (lines 212-216) (This was also addressed in question number 6, raised by reviewer 3)

The authors use thresholds for MASP-3 levels of 5,500 ng/mL and for MAp44 of 2,300 ng/mL, respectively, for their analyses; are these clinically determined or relevant thresholds, or just randomly chosen by the authors?
Answer: We chose the values based on their significance in the Wilcoxon-signed rank distribution test. The thresholds for MASP-3 - 5,500 ng/mL (P<0.0004) and MAp44 - 2,300 ng/mL (P<0,0001) also represent - rounded values of - median levels of MASP-3 and MAp44 respectively, found in controls. We sought then to analyze the frequency of individuals possibly presenting higher levels based on these parameters, once they represented what can be considered normal levels, as found in our study. This information was added to Material and Methods and Results (lines 310, 343-344, 362).

Conclusion:

Reviewer #2: The conclusions are fairly straightforward, however the concluding paragraph ends rather abruptly. While not strictly necessary, I think the paper would benefit from a few more sentences addressing the public health relevance of this study.
Answer: The concluding paragraph was altered in order to end the discussion less abruptly and also provide a better summary of results. Thank you for your suggestion.

Editorial and Data Presentation Modifications

Reviewer #1: - Line 120: there is a repetition of the word "molecules";
Answer: Corrected as requested.

- Line 143: repetition of the word "in";
Answer: Corrected as requested (text was rewritten).

- Methods: the authors could provide information about the study design. Was it a case-control study?
Answer: This is a good point. We re-phrased the first sentence in the Methods section to specify the study design.

- Table 2: the format does not allow the visualization of all the data;
Answer: We apologize for the inconvenience and changed the format.

- Line 528: the word "cornification" could be replaced by classification;
Answer: Corrected as suggested.

- Line 666: the word "resistance' is not written correctly;
Answer: Corrected.

- Figure 6: if possible, improve the resolution for better visualization of the data.
Answer: We improved the resolution of Figure 6 for better visualization.

Reviewer #2: Overall, the Introduction is very long-winded and could be reorganized for better flow.
Answer: Thank you for your suggestion, several alterations were made in the Introduction in order to provide a better flow.

In the first paragraph, an explanation of the spectrum of disease manifestation in case of leprosy, would be appreciated.
Answer: Thank you for this suggestion, the following information was added to the Introduction: "Clinical manifestations depend on the host's genetic polymorphisms and environmental factors that modulate the quality of the immune response, ranging from the multibacillary disabling lepromatous from one end of the clinical spectrum to the paucibacillary tuberculoid form at the other end, with borderline forms in between (Fava et al. 2019)".

This could then lead to how the complement system modulates the course of the disease towards either pole.
Answer: Each clinical pole is strongly associated with the T helper 1 or 2 adaptive immunological response. Unfortunately, the connection of these responses with the functional efficiency of the complement system is much less understood. We added the following sentence to the next paragraph: "There is also strong evidence that intracellular C3 cleavage directs T cell activation towards the Th1 pole (West and Kemper, 2019), which is associated with the paucibacillary presentation of the disease."

The information in Lines 118-139 should be condensed. Again, while I appreciate the thorough introduction to the three MASP1-gene products, the information can be presented in a more concise manner.

Answer: We changed the introduction in order to summarize and update important information.

While talking about the findings of previous studies w.r.t role of complement factors and leprosy susceptibility, the authors should specify whether all the findings are from a particular population (example, the Brazilian population) or from different populations.
Answer: All findings mentioned in the introduction (line 180) were obtained by our group with the same cohort of South Brazilian patients, although the controls may differ. Our analyses were carried out not only with a Brazilian population such as the findings cited in our introduction, but in fact with the same cohort used in those studies. This information was added in the Discussion, thank you for pointing this up.

Formatting of the tables needs to be checked to make sure all text is visible and is not cut off (specifically Table 2 and Table 3).
Answer: Done as suggested.

In general, the manuscript needs to be thoroughly checked for grammatical and typographical errors. Some examples:

Line 523 - Change "Mycobacteria" to "mycobacterial"
Answer: Corrected.

Line 611 – Reference is missing
Answer: Corrected.

Line 665 – Correct spelling of "straightforward"
Answer: Corrected.

Line 666 – Correct spelling of "resistance"
Answer: Corrected.

Line 641 - Missing in-text citation number for Ammitzboll et al. (2013)
Answer: Corrected.

Line 646 - Rephrase "increasing almost twice susceptibility"
Answer: Corrected.

Table S1 - The main text mentions 97 lepromatous cases, but Table S1 mentions 98. Please correct the discrepancy.
Answer: Thank you for drawing our attention to this discrepancy, the number 98 on Table S1 was a typo and was corrected.

The spelling of lepromatous needs to be corrected in the legend.

Answer: Corrected.

The in-text citation style needs to be corrected. Please check the PLoS requirements, which state that "In the text, cite the reference number in square brackets."
Answer: Corrected.

Summary and General Comments

Reviewer #1: The study aims to investigate the role of variants in the MASP1 gene and its products in susceptibility to leprosy. Their results contribute to the understanding of the complexity of the immune response triggered by exposure to Mycobacterium leprae, especially the lectin pathway.

Reviewer #2: In this study, Mendes et al. study the MASP1 gene haplotypes and protein product levels in a cohort of leprosy patients and healthy controls. They show that these haplotypes and protein products, which form part of the complement system, can confer protection/susceptibility to leprosy disease. This seems to be the first study to look at this particular gene and its products in the context of leprosy, as well as in the population under study, although it has been looked at in other mycobacterial diseases such as TB. As such, it adds to our knowledge of the impact of immunogenetics on leprosy disease progression.

Reviewer #3: The authors describe an association study of variants of the MASP1 gene and both leprosy phenotypes and protein expression in a small case-control Brazilian sample. The study is well executed, and results are potentially interesting; however, upon careful reading of the manuscript, a few general issues and a number of specific issues emerge, as follows:

General (major) issues:

1. The writing style adopted is particularly confusing and should be reconsidered. For example, there seems to be no systematic description of the results – the authors seemingly jump from individual marker to 2-marker, 3-marker or 5-marker haplotypic analysis at random, which makes the interpretation of the results very difficult. This also seems to reflect on the key table (table 3, please refer to the next comment) of the study. Also, the manuscript will greatly benefit from a revision by a native English speaker;
Answer: Thank you for your comments and suggestions. The MASP1 genotyping section on Materials and Methods was altered to "MASP1 genotyping and haplotyping". In this section, we added more information concerning analyses with the different haplotypes found. We revised the whole manuscript and several spelling, grammar and syntax mistakes were fixed. We also modified the several sentences at the Results section for a better flow, especially at the "MASP1 polymorphisms and haplotypes associated with leprosy" subsection.

2. Tables 2 and 3 are truncated and impossible to read. Please provide readable versions.
Answer: Corrected for a convenient page presentation.

Specific (minor) issues:

1. The authors used the Ridley & Jopling leprosy classification system; however, some of the tests necessary for the classic R & J protocol described in the original paper, referenced by the authors (such as the Mitsuda test), are no longer available; how did the authors performed the classification? This is critical given that some of the most interesting results come from comparison involving the clinical forms of disease;

Answer: This is a good point and we are happy to provide further explanations. Indeed the Mitsuda test is no longer available, however the diagnoses and classification of leprosy is carried out with an existent correlation of the clinical forms with the Mitsuda test. In Brazil, the classification and diagnosis of the clinical forms of leprosy is done with two approaches. First, with the Rabello classification that conceptualizes the polarization of Leprosy. Second, with the Ridley-Jopling classification, that takes into account Leprosy as a spectrum disease including Lepromatous (Multibacillary), Tuberculoid (Paucibacillary), Borderline and Intermediate. These classifications take into account clinical aspects, histopathology and bacilloscopy. Brazilian health workers are required to enter the clinical characteristics of each leprosy patient into the "SINAN" database (Sistema de Informação de Agravos de Notificação). Each patient file contains information regarding: number and localization of skin patches, neural lesions, eyes, hands and feet disabling degrees, bacilloscopy and histopathology results. Based on these informations, patients are diagnosed into one of the clinical forms. We added this last information to Material and Methods (lines 196-204).

2. In "methods", the control group is described as composed by blood donors; could the authors clarify how matching for socio-economical and geographic background was achieved?

Answer: According to information from the blood bank, the blood donors were inhabitants of Curitiba and surroundings and from a low- to middle socio-economical background, as the patient group, enabling the matching (added to line 219).

3. The authors claim that ethnicity of cases and controls was defined based on "ancestry information" but it is not clear how this has been achieved. The sentence "This means 9%..." (lines 215-219) is obscure, please clarify;

Answer: Ancestry information is based on the origin of first-degree relatives, collected upon patient consultations or from blood bank files. We also clarified the mentioned sentences, thank you for pointing this out (lines 221-228).

4. There is a major difference in age between cases and controls, with the controls being much younger; this could pose a problem for a phenotype such as infection, given that disease risk increases dramatically with age. How does the authors deal with this difference? Shouldn't this be addressed in the discussion?

Answer: Aware of this discrepancy, we addressed this issue with the reduced model of multivariate logistic regression, in order to adjust significant univariate results for associated demographic factors, as age (a factor that might influence protein levels according to previous studies), using STATA v.9.2.

5. The strategy for marker selection adopted have limitations well pointed by the authors in the discussion; why didn't the authors performed complete physical coverage of the candidate gene?

Answer: Our principal limitation for this much better approach, which would also point to possible rare variants influencing susceptibility to the disease, was the cost. Multiplex PCR using sequence-specific primers is a convenient, low-cost-effective strategy, which had been already implemented in our laboratory for other complement genes, by the time we started the project. It has the additional advantage of enabling physical haplotyping (Boldt et al. 2016).

6. Why only a sub-sample was used for the serum concentration assays – in particular, the number of controls is much reduced to almost half (116 out of 214). Is this subsample representative of the total sample described on table 1?

Answer: The serum concentration assays were performed using TRIFMA assays outside of Brazil, during a visit of the first author to Aarhus University. Due to limitations for sample shipping, only a portion of the total sample size was analyzed for protein quantification. However, the subsample is representative of the prevalence of clinical forms and MASP1 genotypes, representing the total described.

7. The authors describe a multivariate logistic regression analysis including several co-variates obtained in previous studies (pages 305-307)? What was the rationale for such strategy?

Answer: The rationale behind was to evaluate independency between associated complement protein levels and polymorphisms previously analyzed in the same setting. However, since these analyses did not change the outcome and we did not discuss the results, there is no reason for this sentence to remain. Therefore, we excluded it from the text.

8. In "Results", table 3 (truncated) seems to present data from the 5 markers individually and for the complete 5-markers haplotypes; however, the text describes results for the 2- and 3-markers haplotypes; it would be much less confusing if table 3 includes all genotypic data, with complete info on allele frequencies for all individual markers, 2-, 3- and 5-markes haplotypes;

Answer: We believe that a table containing the information regarding all SNPs and haplotypes found in our analyses would be considerably heavy. Additionally, many results obtained with haplotypes encompassing the two variants of intron 1, or the three variants of exon 12, were not significant. Therefore, we did not add them to Table 3.

9. The information conveyed between lines 391-396 – in particular, the sentence 'In accordance… disease" is confusing, please clarify.

Answer: We corrected it as follows: *"In accordance with this absence of significant difference, there was no association of MASP1 alleles/haplotypes/genotypes with the lepromatous clinical form of the disease, compared with the group containing the non-lepromatous forms."*

Sincerely yours,

Prof. Dr. Angelica Beate Winter Boldt
Departamento de Genética
Setor de Ciências Biológicas
Universidade Federal do Paraná
Caixa Postal 19071, CEP 81531-980. Curitiba, PR, Brazil.
